# Information bias of social gradients in sickness absence: a comparison of self-report data in the Norwegian Mother and Child Cohort Study (MoBa) and data in national registries

**DOI:** 10.1186/s12889-018-6208-9

**Published:** 2018-11-20

**Authors:** Petter Kristensen, Karina Corbett, Ferdinand A. Mohn, Therese N. Hanvold, Ingrid S. Mehlum

**Affiliations:** 1 Department of Occupational Medicine and Epidemiology, National Institute of Occupational Health, Oslo, Norway; 2 Institute for Social Research, Oslo, Norway

**Keywords:** Data quality, Differential error (misclassification), Education level, Information bias, Pregnancy, Register data, Self-report data, Sickness absence, Social inequalities in health, The Norwegian Mother and Child Cohort Study (MoBa)

## Abstract

**Background:**

Measurement error in self-report questionnaires is a common source of bias in epidemiologic studies. The study aim was to assess information bias of the educational gradient in sickness absence among participants in the Norwegian Mother and Child Cohort Study (MoBa), comparing self-report data with national register data.

**Methods:**

MoBa is a national prospective cohort study. The present study included 49,637 participants, born 1967–1976, who gave birth 2000–2009. The highest completed education level was recorded in categories and as educational years. Sickness absence was defined as one or more spell lasting more than 16 days between pregnancy weeks 13 and 30. We computed sickness absence risk in mid-pregnancy in strata of education level. Associations between completed educational years and sickness absence were estimated as risk differences in binomial regression and compared between self-report and register data. In additional analyses, we aimed to explain discrepancies between estimates from the two data sources.

**Results:**

The overall registry-based sickness absence risk was 0.478 and decreased for increasingly higher education in a consistent fashion, yielding an additive risk difference in association with one additional education year of − 0.032 (95% confidence interval − 0.035 to − 0.030). The self-report risk was lower (0.307) with a corresponding risk difference of only − 0.013 (95% confidence interval − 0.015 to − 0.011). The main explanation of the lower risk difference in the self-report data was a tendency for mothers in low education categories to omit reporting sickness absence in the questionnaire.

**Conclusions:**

A plausible explanation for the biased self-report association is complexity of the sickness absence question and a resulting educational gradient in non-response. As shown for sickness absence in mid-pregnancy in the present study, national registries could be a preferred alternative to self-report questionnaires.

**Electronic supplementary material:**

The online version of this article (10.1186/s12889-018-6208-9) contains supplementary material, which is available to authorized users.

## Background

Measurement error in variables constitutes a fundamental cause of information bias in epidemiology [1]. Data are commonly derived from registries or as self-reports from questionnaires or interviews. Register data are often referred to as secondary, being collected for administrative or other purposes than responding to specific research questions [2]. Because data in different registries usually are collected independently of each other, measurement errors tend to be non-differential, resulting in conservative bias toward the null in studies examining exposure-outcome associations [2]. Self-report data will often be tailor-made, addressing a specific research question, but will be more susceptible to subjective factors. A particular problem could arise when both exposure and outcome depend on self-report data. In this situation, the size of measurement errors in exposures and outcomes could correlate (dependent error) [3]. The resulting information bias is often termed common method bias [4]. Dependent error could result in serious information bias of associations even if the descriptive quality of each separate variable is good [5].

Socioeconomic position refers to «the social and economic factors that influence what positions individuals or groups hold within the structure of a society» [6]. Occupational class has mostly been used as indicator of socioeconomic position (7–16), but education level is an alternative [6, 7]. Three- to six-fold increases in sickness absence across socioeconomic position have been found in Nordic, French and British studies, and in most instances somewhat stronger for men than for women [7–16]. In one study [7], education level and occupational class yielded approximately similar sickness absence gradients.

Data on both socioeconomic position and sickness absence can be obtained as self-reports or from registries. Self-report education level was consistently reported to be higher than census records in a US study [17]. Discrepancies between register and self-report data have also been reported for sickness absence [18–26]. Sickness absence agreement between self-report and administrative or company registries have overall been acceptable but with a tendency of lower self-report reporting [18–26]. Sickness absence in pregnancy was assessed in one study [22], but with limited number of pregnant participants. We are not aware of studies where the main aim has been to compare estimates of social gradients in sickness absence based on self-report and register data.

The Norwegian Mother and Child Cohort Study (MoBa) is a prospective population-based pregnancy cohort study conducted by the Norwegian Institute of Public Health [27]. The validity of MoBa studies has been a matter of concern because of rather low participation [28] and extensive use of maternal self-report data [29–33]. The self-report data in MoBa include sickness absence from work in different parts of the pregnancy. Maternal self-report data in MoBa on drug use, diet, and smoking have been compared with information from national registries [29, 30] and biomarkers [31–33] and suggest acceptable reliability. We are however not aware of MoBa studies addressing information bias of associations, using self-report data on both exposure and outcome.

Norway has several national registries providing demographic, social benefit, or health data that could be helpful in the evaluation of self-report MoBa data. We have established a cohort of all 626,928 persons, live-born in Norway during 1967–1976, with individual linking of data throughout life from several national registries [34]. These data were linked to MoBa data. The main study aim was to assess information bias of the educational gradient in mid-pregnancy sickness absence, comparing self-report data and data in national registries. We examined the results in additional analyses in order to reveal sources of information bias. Because self-report data came from the same source, common method bias and a false under-estimation of the gradient could be a particular concern if mothers were prone to combined under-reporting of sickness absence and over-reporting of education level.

## Methods

### Study population

The MoBa cohort study includes 114,500 children and 95,000 mothers with a main aim to study the causes of disease among mothers and children [35]. It is the largest birth cohort out of a considerable number established worldwide [36]. Participants have been recruited from all over Norway, and 41% of invited women have consented to participate. Follow-up is mainly conducted by questionnaires at regular intervals during pregnancy and after childbirth [37] as well as through the Medical Birth Registry of Norway (MBRN). MoBa mothers were individually linked to the registry-based cohort of all live-born Norwegians 1967–1976 [34] by means of the unique national identification number. Statistics Norway performed the linkage and de-identified the data. In the linkage, 49,637 out of totally 304,945 women in the registry-based cohort were identified as MoBa mothers and constituted the study population. These mothers contributed 59,728 MoBa pregnancies. We used data obtained from each mother’s first MoBa pregnancy.

### Data and variables

We merged data from several sources. MoBa data derive mainly from questionnaires from early pregnancy onwards, biological specimens from the mother and child, and data recorded in MBRN. All questionnaires and detailed instrument documentations can be retrieved at the MoBa website [37]. MoBa questionnaires are extensive, e.g., the first questionnaire numbers 16 pages and contains 144 questions. Some questions are complex, including several details on preselected categories, quantitative responses, and free text. We used MoBa data from Questionnaire 1 (week 15, education) and Questionnaire 3 (week 30, sickness absence) (see Additional file 1: Appendix), as well as a standard MBRN research file of births 2000–2009 to the MoBa mothers [38]. The registry-based cohort included data from MBRN, the national events database (FD-Trygd), and the National Education Database (NUDB). MBRN delivered birth records of the MoBa mothers 1967–1976. FD-Trygd provided daily event data since 1992 on demographic factors and social benefits, including start and stop of employment and sickness absence as well as dates of childbirths [39]. NUDB delivered data on education [40].

#### Education level

NUDB provides annual data on both ongoing and completed education as a 6-digit code where the first digit represents education level [40]. Completed education in the year of MoBa birth was collapsed from nine levels into five categories: Lower secondary or less (level 0–2); Upper secondary, basic (level 3); Upper secondary, complete (levels 4–5); Tertiary, undergraduate (level 6); and Tertiary, graduate (levels 7–8). We also used annual data on ongoing education to compute duration in years registered within each education level. NUDB includes education in Norway in addition to education abroad that is supported financially by the Norwegian State Educational Loan Fund [41].

Questionnaire 1 provided data on the mother’s highest level of completed education (Q50) [37]. The six categories in Q50 are based upon the NUDB standard measures of education level [40]. We merged categories 3 and 4 (technical high school and 3-year high school general studies) into an “Upper secondary, complete” category, identical to the categorization of the register data.

#### Sickness absence in mid-pregnancy

Registry-based and self-report sickness absence were defined as at least one spell lasting more than 16 days between week 13 of pregnancy and the response of Questionnaire 3 (usually in week 30). This was dichotomized as a 0/1 variable. Identification of the calendar dates for week 13 in pregnancy (84 days after last menstruation) and Questionnaire 3 response was only possible after verification of date of giving birth (FD-Trygd); specifying gestational duration (MBRN data in the MoBa file); and specifying the number of days between Questionnaire 3 response and birth (Questionnaire 3). Calendar date for week 13 was computed as date of birth minus gestational duration in days plus 84. Calendar date for Questionnaire 3 response was computed as date of birth minus number of days between the questionnaire response and birth.

We recorded employment in FD-Trygd between the start of pregnancy and Questionnaire 3 response. Mothers registered as wage earner or self-employed were considered to be at risk of sickness absence. FD-Trygd records contain start and stop dates for doctor-certified sickness absence spells lasting more than 16 days. Employees in Norwegian enterprises are fully paid by the employer during certified sickness absence, and the employer is reimbursed by The Norwegian Labour and Welfare Administration for spells exceeding 16 days. Reimbursement also covers self-employed persons. Registration is therefore considered complete. Registration was restricted to the first four spells recorded in FD-Trygd between week 13 and Questionnaire 3 response.

Self-report sickness absence was based on data in Questionnaire 3. We considered all women who reported that they had been in paid employment during the study pregnancy (Q61) to be at risk. These women were asked to respond to questions aimed at surveying sick leave. We used Q75 that included duration of sickness absence from week 13 until completion of Questionnaire 3. A maximum of four spells could be reported. We classified one or more spells lasting more than 16 days as self-report sickness absence; mothers with no reported or shorter spells were classified with no self-report sickness absence. Further details are provided in the Appendix (see Additional file 1).

#### Covariates

We had data on a number of potential confounders, based on prior knowledge on relations to education level and sickness absence [7–16, 42, 43]. MBRN provided mothers’ and maternal grand-mothers’ age when giving birth. FD-Trygd [39] provided data on MoBa mothers’ births before 2000, region of residence 1999, and marital status 1999. NUDB had data on the highest education level to the maternal grandparents when the MoBa mother was 16 years of age. Categories for these covariates are provided in (see Additional file 1: Table S1).

### Analysis

We used Stata/SE 14.1 software (Stata Corporation, College Station, Texas, U.S.A).

#### Main analyses

Education level distributions were tabulated and sickness absence risks in mid-pregnancy were estimated for register and self-report data. We estimated agreement between the two sources for each of the two variables in order to compare register and self-report data.

Levels were also classified as educational years, applying the NUDB norm assigned to each level (9, 11, 12, 15, and 18 years for increasingly higher levels) [40]. Associations between educational years and sickness absence were estimated in binomial regression with sickness absence as dependent variable in separate analyses for register and self-report data. We performed both crude and multivariable analysis, in which we adjusted for potential confounders. Missing covariate data were included as separate categories. Stata’s *binreg, rd* option yielded additive sickness absence risk differences (RD) in association with one extra year of education. Throughout, we included 95% confidence intervals (CI) for the estimated results.

#### Additional analyses

We conducted additional analyses searching for explanations of observed differences between register and self-report data in educational sickness absence gradients. We had main focus on dependent and differential error. Dependent error would be present if measurement errors in education level and sickness absence correlate [3]. Differential error is by definition present if measurement error in the exposure is heterogeneous across true levels of the outcome, or vice versa [3]. This could be the case if completeness of the mother’s sickness absence reporting was dependent on her education level in the NUDB.

In addition to these pre-planned analyses, we carried out two post-hoc analyses. First, we looked at discrepancies in register and self-report education data by examining whether actual years spent on education according to NUDB were similar for mothers who reported the same education level as in the registry, as for mothers who reported higher levels than in the registry. Discrepancies could be due to differences in the participants’ interpretations of Q50 in Questionnaire 1. This was of particular interest for tertiary undergraduates according to the registry who claimed to be tertiary graduates. Second, we carried out sensitivity analyses in order to assess the impact of missing data on education and sickness absence from the two sources. This was mainly done by comparing registry-based educational gradients in participant subsets with and without complete self-report data, as well as self-report gradients in subsets with and without complete register data.

## Results

**Fig. 1:**
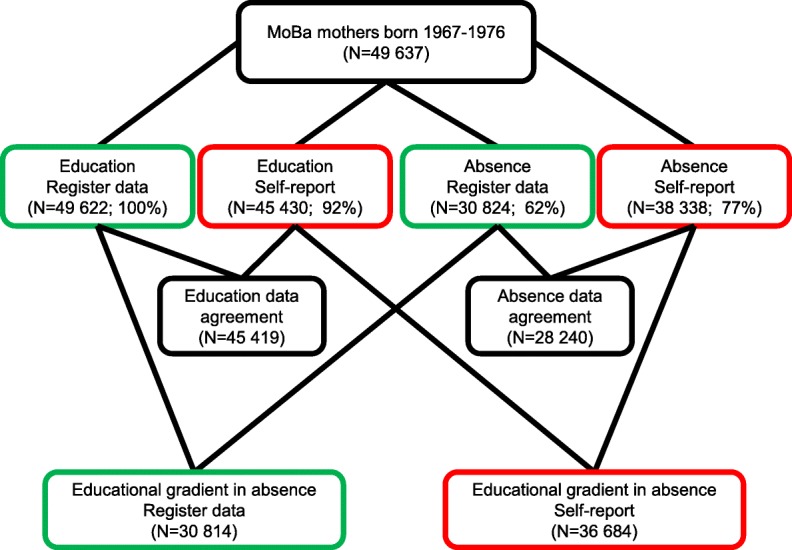
Flow chart of mothers participating in study of educational gradient in sickness absence. Green frame: register data; red frame: self-report data. Exclusions: Education, register data: 15 mothers with missing data. Education, self-report: 4207 mothers because they did not take part in Questionnaire 1 (*N* = 2149) or did not provide valid education level answer (Q50; *N* = 2058). Sickness absence, register data: 8555 missed dates for gestational week 13 or Questionnaire 3 completion, another 2392 had missed information on employment in FD-Trygd, another 20% (7866/41082) had no registry-based employment or self-employment between pregnancy start and Questionnaire 3 completion. Sickness absence, self-report: 4956 did not fill out Questionnaire 3, another 3317 responded to questionnaire version A that did not include duration of sickness absence (Q75), another 7% (3026/41364) did not report job in pregnancy (Q61)

The number of participants in the different analyses is outlined in Fig. 1. Analyses included education level for 49,622 mothers with available register data and 45,430 mothers with self-report data. Sickness absence risk was computed for 30,824 mothers with register data and 38,338 mothers with self-report data. Mothers were more prone to report gainful employment in pregnancy than what was notified in the registry (93% vs. 80%, see Fig. 1 legend). Employment tended to increase with increasingly higher education level, from 75 to 96% for self-report data and from 68 to 83% for register data.

### Main analyses

Table 1 shows that MoBa mothers were highly educated with more than two thirds having completed tertiary education level, both according to register and self-report data. Overall, the distributions were similar, the main difference being that mothers reported more graduate tertiary education and less undergraduate tertiary education than in the registry. Mean educational years was modestly higher for self-report than for register data (14.8 vs. 14.4 years). The sickness absence risk in mid-pregnancy was considerably lower (0.307) in self-report than in register data (0.478). Register data showed a consistent decrease in sickness absence risk for increasingly higher education levels with a risk 0.295 higher in the lowest compared to the highest level. By contrast, self-report sickness absence risk was only moderately lower in tertiary education levels than in lower levels (range between highest and lowest risk 0.080), and the gradient had not nearly the same consistence as for register data.

**Table 1:** Education level distribution and sickness absence risk in mid-pregnancy according to source of data: 49637 MoBa mothers

Source of data and education level	N	%	Sickness absence cases^a^	Risk^a^	95% CI
Register data			14,731	0.478	0.473 to 0.484
Education level (15 missing)					
Lower secondary or less	2431	5	689	0.634	0.605 to 0.663
Upper secondary, basic	1638	3	559	0.630	0.598 to 0.661
Upper secondary, complete	11,484	23	3506	0.539	0.527 to 0.551
Tertiary, undergraduate	25,523	51	8024	0.484	0.476 to 0.492
Tertiary, graduate	8546	17	1953	0.339	0.327 to 0.352
Self-report data			11,279	0.307	0.303 to 0.312
Education level (4207 missing)					
Lower secondary or less	835	2	149	0.336	0.292 to 0.380
Upper secondary, basic	2076	5	455	0.337	0.312 to 0.362
Upper secondary, complete	10,981	24	2758	0.340	0.330 to 0.351
Tertiary, undergraduate	19,779	44	5268	0.318	0.310 to 0.325
Tertiary, graduate	11,759	26	2649	0.260	0.251 to 0.268

*CI* confidence interval

^a^Cases and risks restricted to 30,814 participants with register data and 36,684 participants with self-report data on both education and sickness absence; see Fig. 1

Additional file 1: Table S1 provides distributions of covariates and their relations to educational years and sickness absence risk.

Table 2 shows the distribution of education level according to categories of register data and self-report data. The total agreement was (594 + 523 + 7910 + 17,862 + 7345)/45,419 = 0.75 (95% CI 0.75 to 0.76). Except for the lowest education level, disagreement was mainly restricted to neighbouring categories. Higher self-report level (0.18 of all) was more common than higher register level (0.07 of all). The most frequent disagreement was 4180 (9% of all) who had undergraduate tertiary education recorded in the registry and self-report graduate tertiary level.

**Table 2:** Agreement in education level according to data source: 45419 MoBa mothers. Numbers in cells (fraction of total)

	Self-report data education level					
	Lower secondary or less	Upper secondary, basic	Upper secondary, complete	Tertiary, undergraduate	Tertiary, graduate	Total
Register data education level						
Lower secondary or less	594 (0.013)	829 (0.018)	592 (0.013)	60 (0.001)	10 (0.000)	2085 (0.046)
Upper secondary, basic	19 (0.000)	523 (0.012)	902 (0.020)	33 (0.001)	8 (0.000)	1485 (0.033)
Upper secondary, complete	185 (0.004)	632 (0.014)	7910 (0.174)	1246 (0.027)	214 (0.005)	10,187 (0.224)
Tertiary, undergraduate	31 (0.001)	90 (0.002)	1487 (0.033)	17,862 (0.393)	4180 (0.092)	23,650 (0.521)
Tertiary, graduate	1 (0.000)	2 (0.000)	88 (0.002)	576 (0.013)	7345 (0.162)	8012 (0.176)
Total	830 (0.018)	2076 (0.046)	10,979 (0.242)	19,777 (0.435)	11,757 (0.259)	45,419 (1.000)

Table 3 includes results for sickness absence agreement. Total agreement was 21,138/28240 = 0.75 (95% CI 0.74 to 0.75). Register sickness absence risk was higher than self-report risk, the crude risk difference being 0.17 (95% CI 0.16 to 0.17).

**Table 3:** Agreement in sickness absence according to data source: 28240 MoBa mothers. Numbers in cells (fraction of total)

	Self-report data		
	Not absent	Absent	Total
Register data			
Not absent	13,506 (0.478)	1164 (0.041)	14,670 (0.519)
Absent	5938 (0.210)	7632 (0.270)	13,570 (0.481)
Total	19,444 (0.689)	8796 (0.311)	28,240 (1.000)

The crude RD estimates of mid-pregnancy sickness absence in association with a one-year increment in education were − 0.032 (95% CI –0.035 to − 0.030) for register data and − 0.013 (95% CI –0.015 to − 0.011) for self-report data. Adjustment for potential confounders (see Additional file 1: Table S2) attenuated both RD estimates moderately, to − 0.025 (95% CI –0.028 to − 0.023) and − 0.011 (95% CI –0.013 to − 0.008), respectively.

### Additional analyses

Results of dependent error analyses are presented in Table 4. We computed the distribution of maternal and register data for both education and sickness absence. Overall, the observed distribution was close to the expected on basis of the marginal distributions. There was a weak tendency for clustering for 1139 mothers who, compared with register data, reported higher education level and lower sickness absence (observed fraction 0.0421, expected fraction 0.0365). Excluding the 1139 had only slight impact on RD estimates in the crude regression analysis of self-report associations, with a point estimate change from − 0.013 to − 0.012.

**Table 4:** Discrepancy between register data and self-report data on education level and sickness absence

	Sickness absence			
	Maternal absence, no register absence	Maternal and register data agreement	No maternal absence, register absence	Total
Education level				
Maternal level higher than register level	*N* = 188	*N* = 3397	*N* = 1139	
	O = 0.0070	O = 0.1256	O = 0.0421	*N* = 4724
	E = 0.0072	E = 0.1310	E = 0.0365	O = 0.1747
Maternal and register data agreement	*N* = 869	*N* = 15,799	*N* = 4156	
	O = 0.0321	O = 0.5842	O = 0.1537	*N* = 20,824
	E = 0.0318	E = 0.5773	E = 0.1610	O = 0.7701
Maternal level lower than register level	*N* = 59	*N* = 1075	*N* = 360	
	O = 0.0022	O = 0.0398	O = 0.0133	*N* = 1494
	E = 0.0023	E = 0.0413	E = 0.0115	O = 0.0552
Total	*N* = 1116	N = 20,271	*N* = 5655	*N* = 27,042
	O = 0.0413	O = 0.7496	O = 0.2091	O = 1.000

*N* Number in category, *O* Observed fraction, *E* Expected fraction assuming independence. (E.g., expected frequency for mothers higher on education and lower on sickness absence = 0.1747 × 0.2091 = 0.0365)

An assessment of differential error is provided in Table 5. Here, both self-report and registry-based sickness absence was examined in association with the assumingly correct NUDB educational attainment and compared. The educational gradient in self-report sickness absence risk (0.397–0.246 = 0.151; column A) was half the size the gradient based only on register data (0.663–0.343 = 0.320; column B). A differential pattern is evident: the additive difference between the two sources of sickness absence was nearly threefold higher in the lowest compared to the highest education level (0.27 vs 0.10).

**Table 5:** Sickness absence risk according to source of information, stratified by education level in NUDB^a^

Education level in NUDB	Sickness absence risk		
	A: Self-report	B: Register data	Difference B-A (95% CI)
Lower secondary or less	0.397	0.663	0.27 (0.23 to 0.30)
Upper secondary, basic	0.368	0.638	0.27 (0.23 to 0.31)
Upper secondary, complete	0.339	0.541	0.20 (0.19 to 0.22)
Tertiary, undergraduate	0.316	0.487	0.17 (0.16 to 0.18)
Tertiary, graduate	0.246	0.343	0.10 (0.09 to 0.11)

*CI* Confidence interval, *NUDB* National Education Database

^a^Analysis restricted to 28,233 MoBa mothers with register data on both education and sickness absence, and self-report data on sickness absence

The most evident discrepancy in education level was 4180 undergraduates according to NUDB (9.2% of all participants) who reported graduate level (Table 2). The median duration of tertiary undergraduate education in the registry for the 17,862 mothers with agreement on completed undergraduate level in the two sources was four years, and 35% spent more than four years conducting their undergraduate studies. The 4180 undergraduates according to the registry who reported graduate level were slower in completing their studies: undergraduate studies had a median duration of five years and 64% on this level studied for more than four years.

Missing data mainly affected register sickness absence and self-report education (Fig. 1). The sensitivity analysis revealed that self-report sickness absence was slightly lower (0.291) among mothers with missing register sickness absence data compared to mothers with such data (0.311). Mean educational years in NUDB were lower (13.8) among mothers missing self-report education data than among mothers who had reported their education level (14.5). Source-specific educational gradients in subsets according to availability of data from the other source were consistent: the register data gradients were throughout more than twice as strong as the self-report gradients (Additional file 1: Table S3).

## Discussion

Mothers participating in MoBa tended to report somewhat higher education level and considerably less sickness absence in mid-pregnancy compared with data in national registries. Educational attainment was negatively associated with sickness absence. This association was considerably stronger for register data than for self-report data.

### Strengths and weaknesses

MoBa is a large and population-based prospective study with an extensive and detailed documentation of available data in the questionnaires. The Norwegian national registries used in this study are considered complete for residents. Registrations of education in NUDB and sickness absence in FD-Trygd are based on administrative notifications, independent of each other and independent of maternal self-report. Individual linkage between different data sources is feasible due to the national identification number.

Register data could be considered correct for education but could be more problematic for sickness absence. NUDB data are based on reports from educational institutions in Norway and abroad, and we assessed the criterion validity to be reasonably high. Assessment of mid-pregnancy sickness absence in FD-Trygd is more complex. One important issue in our study is that sickness absence ascertainment in the registry was dependent on correct dates for week 13 in pregnancy and Questionnaire 3 response. A relatively large proportion was excluded from analysis because of missing timing of pregnancy start and the response of Questionnaire 3. If mothers missing information on timing were prone to low education level and high sickness absence risk in mid-pregnancy the most plausible problem would be an underestimation of the educational gradient in sickness absence for both self-report and register data. The sensitivity analysis (Additional file 1: Table S3) suggests that the different pattern in register and self-report gradients was not critically dependent on missing information.

### Comparison with other studies and inferences

Reports of self-report overestimation of education level [17] and underestimation of sickness absence [18–26] are in agreement with the results in the present study. However, our main objective was not to explore reliability but rather to explore educational gradients in sickness absence and to seek explanations for different gradients in data from the two sources.

We suspected dependent error [3, 5] resulting in common method bias [4] in the self-report estimate. Errors in self-reports of the two variables did however not correlate (Table 4), resulting in minimal effect on the association. One reason for this lack of dependent error could be due to the relative objectiveness of the education and sickness absence variables, with neither of the two being dependent on respondent sentiment or personality.

Rather few mothers in the low education categories reported sickness absence. This turned out to be the main explanation of the weaker gradient compared to the registry-based analysis. We have no data to explain this, but the complexity of Q75 could be one reason. Q75 consists of several detailed elements for up to four spells (see Additional file 1: Appendix). The weak self-report gradient could arise from a lack of completion of Q75, if this problem was more common among the lowly educated. Another possible explanation could be that the self-report at-risk criterion was wide and included participants who were not entitled to sickness absence benefit. Ninety-three percent reported a job in pregnancy, which was higher than in the register data (Fig. 1 legend), and higher than what should be expected from national statistics [44]. This could have deflated the overall self-report sickness absence risk but is not a likely explanation of the weak gradient because the higher self-report work attendance was primarily a characteristic of the highly educated.

Several questions in MoBa questionnaires have the same structure and complexity as Q75 in Questionnaire 3 [37]. One example is drug use in pregnancy [37]. Skurtveit et al. [30] have compared MoBa mothers’ self-report and data in the Norwegian Prescription Database on drug use during pregnancy. They show that agreement between the two sources varied for different drug types ([30]; Table 1). Database registered use of opioids and benzodiazepine anxiolytics, not reported by participants, constituted a considerable proportion of the total number of users among MoBa mothers. Although reasons for maternal lack of drug use response could be different from lack of sickness absence response in our study, it could be interesting to explore if drug non-response showed the same educational gradient.

The high number of tertiary undergraduates according to the registry, who reported to be graduates could partly be due to a misunderstanding of questions, as seen in a U.S. study [17]. The Questionnaire 1 wording “more than 4 years” (see Additional file 1: Appendix) was evidently meant as the normative duration of tertiary education and classified accordingly in NUDB. A portion of the MoBa mothers could have interpreted this as years spent in education. The duration of ongoing undergraduate tertiary education among those with NUDB data agreement and the 4180 that considered themselves graduates supports this explanation.

The results of this study are highly dependent on conduct and details in the MoBa questionnaires and cannot be generalized to be true for all similar studies. The lesson learnt is rather that complexity and details of questionnaires could be important for the internal validity of estimated associations.

## Conclusions

MoBa is an example that birth cohorts can be excellent sources of the scientific study of a multitude of health issues relating to parents and children [45]. Maternal self-report data are widely available in MoBa questionnaires and provide an opportunity for studies solely based on self-report exposure and outcome data, including maternal education level [46, 47] and absence from work in pregnancy [48]. MoBa questionnaires include some complex and time-consuming items, and the possibility and potential consequences of missing and inconsistent responses should therefore be scrutinized. This problem resulted in differential error and a biased underestimation of the educational gradient in mid-pregnancy sickness absence risk. Common method bias was apparently not a problem in our study, but could jeopardize validity in studies addressing topics with questions more heavily influenced by maternal trait and personality. Norway has excellent national demographic, social, and health registries offering alternative data that might solve such problems. National registries could be an alternative data source, similar to the Danish National Birth Cohort [49]. Finally, the apparent misinterpretation by some participants when responding the education level question reminds us of the importance of wording details in questionnaires.

## Additional file

Additional file 1: Appendix. MoBa questionnaire text. **Table S1.** Covariate distribution and relation to mean years of education and sickness absence risk. **Table S2.** Associations between years of education and sickness absence according to data source. **Table S3.** Educational gradient in sickness absence risk according to data source and completeness of data. (DOCX 33 kb)

## Abbreviations

CI: confidence interval
MBRN: Medical Birth Registry of Norway
MoBa: Norwegian Mother and Child Cohort Study
NUDB: National Education Database
RD: risk difference
