# Supplementary material for: Information bias of social gradients in sickness absence: a comparison of self-report data in the Norwegian Mother and Child Cohort Study (MoBa) and data in national registries
Source: BMC Public Health. 2018 Nov 20;18:1275. doi: 10.1186/s12889-018-6208-9 (PMC6245919; doi:10.1186/s12889-018-6208-9)
Supplement: Supplementary file 1 — Appendix. MoBa questionnaire text. Table S1. Covariate distribution and relation to mean years of education and sickness absence risk. Table S2. Associations between years of education and sickness absence according to data source. Table S3. Educational gradient in sickness absence risk according to data source and completeness of data. (DOCX 33 kb) [file 12889_2018_6208_MOESM1_ESM.docx]

**Information bias of social gradients in sickness absence: a comparison of self-report data in The Norwegian Mother and Child Cohort Study (MoBa) and data in national registries**

**Additional file**

Petter Kristensen^1^, Karina Corbett^1^, Ferdinand A Mohn^1,2^, Therese N Hanvold^1^, Ingrid S Mehlum^1^.

^1^ Department of Occupational Medicine and Epidemiology, National Institute of Occupational Health, Oslo, Norway

^2^ Institute for Social Research, Oslo, Norway

Corresponding author: Petter Kristensen, e-mail [pkr@stami.no](mailto:pkr@stami.no)

**Contents**

|  | Page |
| --- | --- |
| Appendix | 2 |
| Table S1 | 3 |
| Table S2 | 4 |
| Table S3 | 5 |

**Appendix**

The current study was based on version 6 of the quality-assured MoBa data files released for research. Questions in Questionnaire 1 and Questionnaire 3 relate to education level and sickness absence, respectively. Details below are extracted from MoBa Questionnaires and Instrument documentations, to be found at . <https://www.fhi.no/en/studies/moba/for-forskere-artikler/questionnaires-from-moba/>

**Questionnaire 1**

**Rationale for choosing Q50:** These are standard measures of education levels.

**Q50** What education do you and the baby’s father have? (Enter a cross indicating the highest level of education you both have completed and current studies if you are still studying)^a^

1 9-year secondary school

2 1-2 year high school

3 Technical high school

4 3-year high school general studies, junior college

5 Regional technical college, 4-year university degree (Bachelor’s degree, nurse, teacher, engineer)

6 University, technical college, more than 4 years (Master’s degree, medical doctor, PhD)

7 Other education

**Questionnaire 3^b^**

**Q61** Have you been in paid employment during this pregnancy? (No/Yes).

**Q75** Complete the table below if you were on sick leave (full or part time) after the 13^th^ week of pregnancy. Fill in the reason (e.g. pelvic girdle pain, pneumonia), which weeks you were on sick leave, the number of days and the percentage of time each period of sick leave represents. (*Give one reason for sick leave per line*).^c^

**Legends:**

^a^ Only the mother’s own completed education was considered. Categories 3 and 4 were collapsed and category 7 classified as missing

^b^ Version A of Questionnaire 3 (1999-2001) did not include duration of sick leave in Q75. Therefore, only the more recent versions B, C, and E (2001-2009) could be applied

^c^ A maximum of four spells could be recorded. Sick leave timing (gestational week) could be recorded in five categories (W13-W16, W17-W20, W21-24, W25-28, W29+)

**Table S1** Covariate distribution and relation to mean years of education and sickness absence risk

| Category | N | % | Register data mean years of education | Self-report mean years of education | Register data absence risk | Self-report absence risk |
| --- | --- | --- | --- | --- | --- | --- |
| Year of MoBa birth | | | | | | |
| 2000-2002 | 23 242 | 47 | 14.1 | 14.4 | 0.516 | 0.332 |
| 2003-2005 | 16 947 | 34 | 14.5 | 14.9 | 0.461 | 0.295 |
| 2006-2009 | 9392 | 19 | 14.9 | 15.4 | 0.430 | 0.275 |
| Missing | 56 | 0 | 13.8 | 14.3 | - | 0.091 |
| Mother’s age when giving MoBa birth (years) | | | | | | |
| 20-29 | 8300 | 17 | 13.9 | 14.0 | 0.480 | 0.313 |
| 30-34 | 28 958 | 58 | 14.5 | 14.9 | 0.476 | 0.307 |
| 35-39 | 11 763 | 24 | 14.5 | 15.0 | 0.483 | 0.301 |
| 40+ | 427 | 1 | 14.8 | 15.4 | 0.496 | 0.299 |
| Missing | 189 | 0 | 14.4 | 14.9 | 0.478 | 0.273 |
| Reproductive history | | | | | | |
| No birth before 2000 | 36 392 | 73 | 14.8 | 15.2 | 0.451 | 0.294 |
| Birth(s) before 2000 | 13 245 | 27 | 13.4 | 13.7 | 0.566 | 0.346 |
| Region of residence 1999 | | | | | | |
| South-East | 22 271 | 45 | 14.5 | 15.0 | 0.501 | 0.318 |
| South | 3688 | 7 | 14.2 | 14.5 | 0.491 | 0.278 |
| West | 16 264 | 33 | 14.3 | 14.5 | 0.433 | 0.282 |
| Mid (Trøndelag) | 3879 | 8 | 14.4 | 14.7 | 0.466 | 0.306 |
| North | 3535 | 7 | 14.3 | 14.6 | 0.532 | 0.356 |
| Residence in 5 major cities | | | | | | |
| No | 34 420 | 69 | 14.2 | 14.5 | 0.493 | 0.306 |
| Yes | 15 217 | 31 | 14.9 | 15.4 | 0.444 | 0.306 |
| Marital status (1999) | | | | | | |
| Married/partnership | 23 912 | 48 | 14.2 | 14.6 | 0.483 | 0.309 |
| Other | 25 177 | 51 | 14.6 | 14.9 | 0.473 | 0.304 |
| Missing | 548 | 1 | 15.3 | 15.7 | 0.460 | 0.297 |
| Maternal grandmother’s age when giving birth to MoBa mother (years) | | | | | | |
| <20 | 3121 | 6 | 13.4 | 13.7 | 0.529 | 0.332 |
| 20-29 | 34 423 | 69 | 14.4 | 14.8 | 0.484 | 0.311 |
| 30-34 | 7920 | 16 | 14.6 | 15.0 | 0.445 | 0.291 |
| 35-39 | 3174 | 6 | 14.5 | 14.9 | 0.463 | 0.281 |
| 40+ | 999 | 2 | 14.5 | 14.8 | 0.421 | 0.260 |
| Maternal grandparents’ highest education | | | | | | |
| Lower secondary or less | 4345 | 9 | 12.9 | 13.1 | 0.551 | 0.320 |
| Upper secondary, basic | 17 885 | 36 | 13.8 | 14.1 | 0.506 | 0.310 |
| Upper secondary, complete | 10 330 | 21 | 14.2 | 14.6 | 0.483 | 0.315 |
| Tertiary, undergraduate | 11 856 | 24 | 15.2 | 15.6 | 0.444 | 0.295 |
| Tertiary, graduate | 5122 | 10 | 16.1 | 16.5 | 0.399 | 0.289 |
| Missing | 99 | 0 | 14.8 | 15.2 | 0.500 | 0.314 |

**Table S2** Associations between years of education and sickness absence according to data source

| Independent variables | Register data (N=30 814) | |  | Self-report data (N=36 684) | |
| --- | --- | --- | --- | --- | --- |
|  | RD | 95% CI |  | RD | 95% CI |
| Years of education | –0.025 | –0.028 to –0.023 |  | –0.011 | –0.013 to –0.008 |
| Year of MoBa birth | | | | | |
| 2000-2002 | +0.045 | +0.032 to +0.057 |  | +0.036 | +0.025 to +0.046 |
| 2003-2005 | 0 | Reference |  | 0 | Reference |
| 2006-2009 | –0.018 | –0.034 to –0.003 |  | –0.012 | –0.025 to +0.001 |
| Mother’s age when giving MoBa birth (years) | | | | | |
| 20-29 | –0.013 | –0.029 to +0.003 |  | +0.000 | –0.014 to +0.015 |
| 30-34 | 0 | Reference |  | 0 | Reference |
| 35-39 | –0.005 | –0.019 to +0.009 |  | –0.011 | –0.022 to +0.001 |
| 40+ | +0.029 | –0.033 to +0.091 |  | –0.005 | –0.056 to +0.046 |
| Missing | +0.007 | –0.194 to +0.207 |  | –0.022 | –0.119 to +0.075 |
| Reproductive history | | | | | |
| No birth before 2000 | 0 | Reference |  | 0 | Reference |
| Birth(s) before 2000 | +0.083 | +0.068 to +0.098 |  | +0.042 | +0.029 to +0.055 |
| Region of residence 1999 | | | | | |
| South-East | 0 | Reference |  | 0 | Reference |
| South | –0.024 | –0.046 to –0.002 |  | –0.041 | –0.059 to –0.023 |
| West | –0.091 | –0.104 to –0.078 |  | –0.047 | –0.058 to –0.036 |
| Mid (Trøndelag) | –0.051 | –0.073 to –0.030 |  | –0.022 | –0.040 to –0.003 |
| North | +0.005 | –0.018 to +0.027 |  | +0.028 | +0.008 to +0.047 |
| Residence in 5 major cities | | | | | |
| No | 0 | Reference |  | 0 | Reference |
| Yes | –0.004 | –0.016 to +0.009 |  | +0.022 | +0.011 to +0.033 |
| Marital status (1999) | | | | | |
| Married/partnership | 0 | Reference |  | 0 | Reference |
| Other | –0.015 | –0.026 to –0.003 |  | –0.007 | –0.017 to +0.004 |
| Missing | +0.010 | –0.043 to +0.063 |  | +0.007 | –0.037 to +0.051 |
| Maternal grandmother’s age when giving birth to MoBa mother (years) | | | | | |
| <20 | +0.005 | –0.019 to +0.029 |  | +0.006 | –0.015 to +0.026 |
| 20-29 | 0 | Reference |  | 0 | Reference |
| 30-34 | –0.026 | –0.041 to –0.011 |  | –0.015 | –0.028 to –0.003 |
| 35-39 | –0.015 | –0.038 to +0.008 |  | –0.028 | –0.047 to –0.009 |
| 40+ | –0.055 | –0.094 to –0.016 |  | –0.048 | –0.080 to –0.016 |
| Maternal grandparents’ highest education | | | | | |
| Lower secondary or less | +0.023 | +0.000 to +0.046 |  | –0.009 | –0.029 to +0.011 |
| Upper secondary, incomplete | +0.010 | –0.005 to +0.025 |  | –0.010 | –0.023 to +0.003 |
| Upper secondary, complete | 0 | Reference |  | 0 | Reference |
| Tertiary, short | –0.009 | –0.026 to +0.007 |  | –0.007 | –0.021 to +0.007 |
| Tertiary, long | –0.028 | –0.049 to –0.007 |  | –0.003 | –0.021 to +0.015 |
| Missing | +0.047 | –0.079 to +0.172 |  | +0.026 | –0.085 to +0.137 |

CI: confidence interval; RD: risk difference

**Table S3** Educational gradient in sickness absence risk according to data source and completeness of data

| Category | Number | Risk difference | 95% CI |
| --- | --- | --- | --- |
|  |  |  |  |
| Register data | | | |
| Self-report data complete | 27 042 | –0.034 | –0.037 to –0.032 |
| Self-report data not complete | 3772 | –0.028 | –0.035 to –0.022 |
| Self-report data | | | |
| Register data complete | 27 042 | –0.016 | –0.018 to –0.014 |
| Register data not complete | 9642 | –0.005 | –0.009 to –0.001 |

CI: Confidence interval
